# Finite Element Analysis of Single Cell Stiffness Measurements Using PZT-Integrated Buckling Nanoneedles

**DOI:** 10.3390/s17010014

**Published:** 2016-12-23

**Authors:** Maryam Alsadat Rad, Auwal Shehu Tijjani, Mohd Ridzuan Ahmad, Shehu Muhammad Auwal

**Affiliations:** Department of Control and Mechatronics Engineering, Faculty of Electrical Engineering, Universiti Teknologi Malaysia, 81310 Skudai, Johor, Malaysia; maryam.pd@utm.my (M.A.R.); atshehu1776@gmail.com (A.S.T.); fancymask01@gmail.com (S.M.A.)

**Keywords:** global stiffness, local stiffness, buckling nanoneedle, PZT-integrated, single cell analysis

## Abstract

This paper proposes a new technique for real-time single cell stiffness measurement using lead zirconate titanate (PZT)-integrated buckling nanoneedles. The PZT and the buckling part of the nanoneedle have been modelled and validated using the ABAQUS software. The two parts are integrated together to function as a single unit. After calibration, the stiffness, Young’s modulus, Poisson’s ratio and sensitivity of the PZT-integrated buckling nanoneedle have been determined to be 0.7100 N·m^−1^, 123.4700 GPa, 0.3000 and 0.0693 V·m·N^−1^, respectively. Three *Saccharomyces cerevisiae* cells have been modelled and validated based on compression tests. The average global stiffness and Young’s modulus of the cells are determined to be 10.8867 ± 0.0094 N·m^−1^ and 110.7033 ± 0.0081 MPa, respectively. The nanoneedle and the cell have been assembled to measure the local stiffness of the single *Saccharomyces cerevisiae* cells The local stiffness, Young’s modulus and PZT output voltage of the three different size Saccharomyces cerevisiae have been determined at different environmental conditions. We investigated that, at low temperature the stiffness value is low to adapt to the change in the environmental condition. As a result, *Saccharomyces cerevisiae* becomes vulnerable to viral and bacterial attacks. Therefore, the proposed technique will serve as a quick and accurate process to diagnose diseases at early stage in a cell for effective treatment.

## 1. Introduction

Cells are the basic structural and functional units of life of all living organisms [1]. To enhance the quality of life, a comprehensive study of cell behavior is pre-requisite [2]. In recent years, there exists considerable progress in the research area on how biophysical and biomechanical behaviors of a cell are affected by diseases such as cancer, diabetes, asthma and malaria [3]. Indeed, physiological changes that take place within a cell coexist with mechanical and physical changes, which are of the utmost importance for the cell to adapt to any changes in environmental condition [3]. This provides room to analyze the mechanical and physical properties of a cells [4]. 

Powerful techniques in molecular biology exist for cells analysis. However, in numerous fields like cancer biology and tissue engineering, results from analysis of average signals from numerous cells may not provide the correct information, which may lead to the wrong conclusions [5]. This is because the behavior of the cells of interest in the sample, which could be less in number, may be masked by the majority or the behavior of the cells of interest changes with time. For this reason, accurate and reliable characterization of cellular samples with high degree of uniformity may only be possible by analyzing single cell. As a matter of fact, changes in the mechanical properties for example, elastic modulus and stiffness [6,7] of a single cell result in the physiological changes [1]. As a result, single cell stiffness measurement can be used to diagnosis numerous dangerous diseases including cancer, diabetes and malaria which changes the physiological behavior of a single cell [5]. Because of this considerable information derived for better understanding of disease diagnosis, infections and drugs response [8] at cell level, single cell stiffness measurement is of great interest. Conversely, the mechanics of cell wall/membrane, which contribute immensely for the measurement of the stiffness of a single cell is an area of “near-total darkness” [9]. 

Conventional procedure used to measure the stiffness of a material cannot be directly applied to the structures at micro and nano level, like biological cells and nanofibres. To determine the mechanical properties of a single cell accurately without causing considerable damage to the cell, a suitable nano force sensor such as a nanoneedle is required. Nanoneedles can be fabricated at a various uneven locations with suitable orientation in atomic force microscopy (AFM) [8]. Nanoneedles can be either rigid or soft. Soft nanoneedles are those that buckle on contact with a stiff surface when a force is applied to them, while on the other hand rigid nanoneedles are strong and can penetrate soft materials. Rigid nanoneedles are not preferred as nanoforce sensors for single cell stiffness measurements, because they cause severe damage to the cells. For this reason, buckling nanoneedles are the solution, despite the fact that measurement of nano Newton forces using buckling nanoneedles requires special instrumentation [10]. 

In the literature, many nanoneedles for single cell stiffness measurement have been proposed and several attempts were made to fabricate nanoneedles that can measure the stiffness of a single cell in real time. Nanoneedles with a buffering beam have been proposed in [5] to measure the stiffness of a single cell with the aid of a nanomanipulator [6] inside an environmental scanning electron microscope (ESEM). In [11,12], rigid nanoneedles were proposed for single cell surgery. The main drawback of this technique is the difficulty of controlling the vibration of the actuator. In [8] a thermistor-based nanoneedle for single cell thermal characterization was investigated. The weakness of this technique is that, there is no absolute control in the speed of the cell in the microfluidic system and the sensing part. In [4], a buckling nanoneedle for single cell stiffness characterization was proposed. The nanoneedle was fabricated by an etching process on the AFM cantilever [13]. In the work of the authors in [3], the effect of temperature on the stiffness and Young’s modulus of three human cancerous cells (MCF-7, HeLa and A549) was investigated. The technique utilizes AFM to indent six different types of human cancer cells under controlled temperature conditions. The image of the indentation was processed in order to investigate the effect of change in temperature on the stiffness and Young modulus of human cancer cells [14]. All the aforementioned techniques for single cell stiffness measurement cannot provide real-time data measurements [4]. Their accuracy depends on the image processing algorithm used, which is relatively slow. In fact, the techniques cause significant damage to the cells under investigation. Furthermore, some techniques are not applicable to some cell categories. As a result, the techniques cannot be used to measure the stiffness of single cells in general. 

In this paper, we propose a lead zirconate titanate (PZT)-integrated buckling nanoneedle for single cell stiffness measurements. The proposed technique is fast, as the PZT sensor generates an output voltage when an input force is applied without transportation delay. Furthermore, the technique is accurate due to the circular flat tip of the nanoneedle that enhances the result using the Hertz-Sneddon mechanics model. This technique, relative to other techniques, provides real-time stiffness measurements at a single cell level. In addition, the buckling phenomena of the nanoneedle prevent cell damage during the indentation process. The technique can also be used to measure the stiffness of different cell categories. As a result, this technique will serve as a quick and accurate process to diagnose diseases at early stage in a single cell for effective treatment. The technique can supplement other present-day biochemical techniques for disease diagnosis, for example regenerative diseases like cancer. The technique could be a great contribution to the field of medicine, biomedical engineering, pharmacy, agriculture and biotechnology. 

## 2. Finite Element Modelling of the PZT-Integrated Buckling Nanoneedle

The most famous technique used for modelling, simulation and validation of complex biomedical engineering, biomedical science and biotechnology analysis is finite element (FE) analysis [9]. ABAQUS 6.14 CAE/CEL is one of the commercially available FE analysis software packages. Because of the flexibility of ABAQUS it was chosen for numerical cell stiffness measurement to validate the experimental data. It provides approximate solutions of complex equations during FE analysis. The proposed different types of nanoneedles include buckling nanoneedles to reduce the cell damage effects during analysis. In this paper, nanoneedles integrated with PZT sensors are used for real-time single cell stiffness measurements. The modelling consists of two parts.

### 2.1. Finite Element Modelling of the PZT Sensor

The sensor part of the buckling nanoneedle works based on a phenomenon known as piezoelectricity. The sensor produces an output voltage proportional to the stiffness of the cell. The output voltage generated is associated with asymmetrical crystalline nature of the PZT material [15,16]. Because of mechanical stresses such as pulling, pressure, torsion, etc., transmitted through the PZT, electric charges accumulate at the surface of the PZT [16,17,18,19]. Conversely, the application of external electric field results in a production of a proportional mechanical deformation [16,19]. PZT devices have been modeled using the finite element method [20,21,22,23]. Moreover, at a microscopic level the piezoelectric effect is produced as result of ionic charge displacement within the asymmetric crystalline material. Conversely, a symmetric crystalline material does not exhibit piezoelectricity [15]. Actually, piezoelectric sensors are more suitable for changing mechanical quantities into the corresponding electrical quantities or signals, because of their sensitivity to slight variations in force, pressure (stress) and vibration [16]. Piezoelectric sensors can be categorized into two classes:

- Axial sensors, where the force or stress is applied along the polarization axis, called 33-mode.
- Bending sensors, where the force or stress is applied at right angle to the polarization axis, called 31-mode.

**Figure 1:**
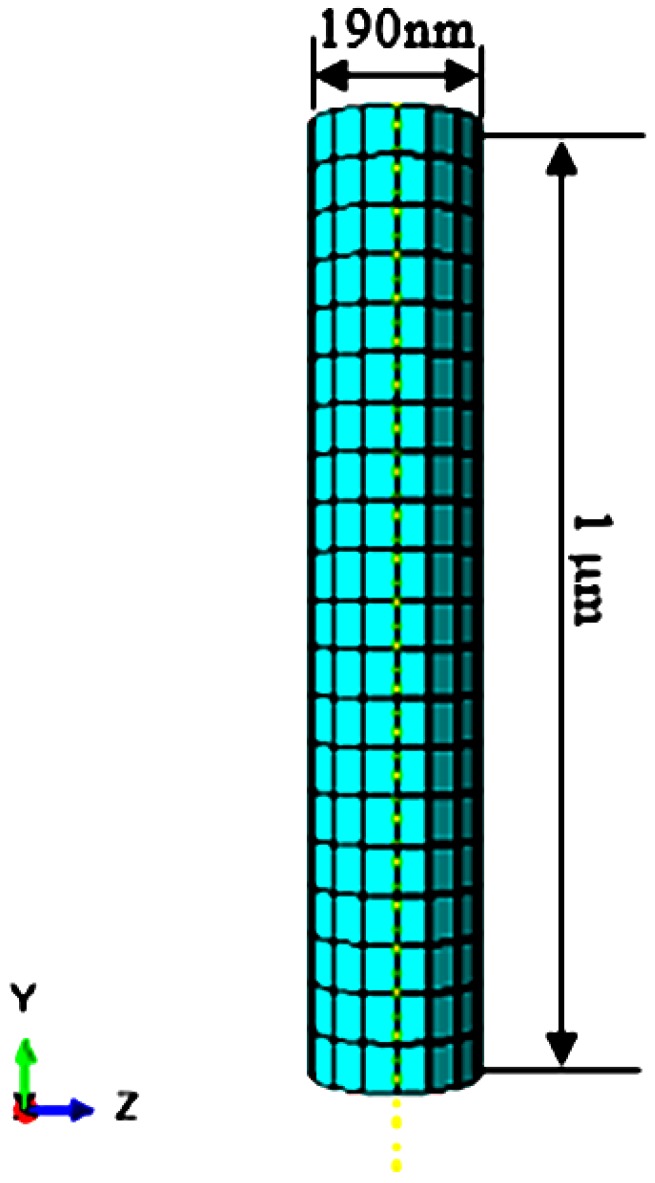
PZT sensing part of the buckling nanoneedle.

In this paper, we utilized a direct piezoelectric effect in the sensing part of the PZT-integrated buckling nanoneedle. The sensing part was designed with cylindrical geometry. This part of the PZT-integrated buckling nanoneedle will be in contact with the cell for which its stiffness is to be measured. As a result, the cell’s Young’s modulus can be computed accurately using the Hertz-Sneddon mechanics model [4]. The PZT part has a radius of 95 nm and height of 1 µm, as shows in Figure 1. Units of consistency were used in the ABAQUS. As a result, all the dimensions used in modelling are based on the micro-MKS system. For this reason, the true behavior of the model at a micro level was investigated without using any scaling factor. The piezoelectric material modelled is based on PZT-5H [24] having the piezoelectric, elastic and permittivity features given in Table 1. These PZT sensor properties were defined during the FE modelling process. In SI units, the density of the PZT-5H material is 7500 kg·m^−3^, equivalent to 7.5 × 10^−15^ kg·μm^−3^ in the micro-MKS system.

### 2.2. Electric Potential Analysis of the PZT Sensor

In tensor and vector form, the general piezoelectric constitutive equation for piezoelectric sensor and actuator is given by Equation (1) [25]:

(1) $$\left(\begin{array}{c} D_{1}\\ D_{2}\\ D_{3} \end{array}\right)=\left(\begin{array}{cccccc} d_{11} & d_{12} & d_{13} & d_{14} & d_{15} & d_{16}\\ d_{21} & d_{22} & d_{23} & d_{24} & d_{25} & d_{26}\\ d_{31} & d_{32} & d_{33} & d_{34} & d_{35} & d_{36} \end{array}\right)\left(\begin{array}{c} T_{1}\\ T_{2}\\ T_{3}\\ T_{4}\\ T_{5}\\ T_{6} \end{array}\right)+\left(\begin{array}{ccc} \varepsilon_{11} & \varepsilon_{12} & \varepsilon_{13}\\ \varepsilon_{21} & \varepsilon_{22} & \varepsilon_{23}\\ \varepsilon_{31} & \varepsilon_{32} & \varepsilon_{33} \end{array}\right)\left(\begin{array}{c} E_{1}\\ E_{2}\\ E_{3} \end{array}\right)$$

where, *D*, *d*, *ε* and *E* are the electrical polarization vector, piezoelectric coefficient matrix, stress vector, electrical permittivity matrix and electrical field vector, respectively.

Equation (1) is applicable to the PZT part when the external electric potential, *E*, is zero. Hence, Equation (1) becomes:

(2) $$\left(\begin{array}{c} D_{1}\\ D_{2}\\ D_{3} \end{array}\right)=\left(\begin{array}{cccccc} d_{11} & d_{12} & d_{13} & d_{14} & d_{15} & d_{16}\\ d_{21} & d_{22} & d_{23} & d_{24} & d_{25} & d_{26}\\ d_{31} & d_{32} & d_{33} & d_{34} & d_{35} & d_{36} \end{array}\right)\left(\begin{array}{c} T_{1}\\ T_{2}\\ T_{3}\\ T_{4}\\ T_{5}\\ T_{6} \end{array}\right)$$

**Figure 2:**
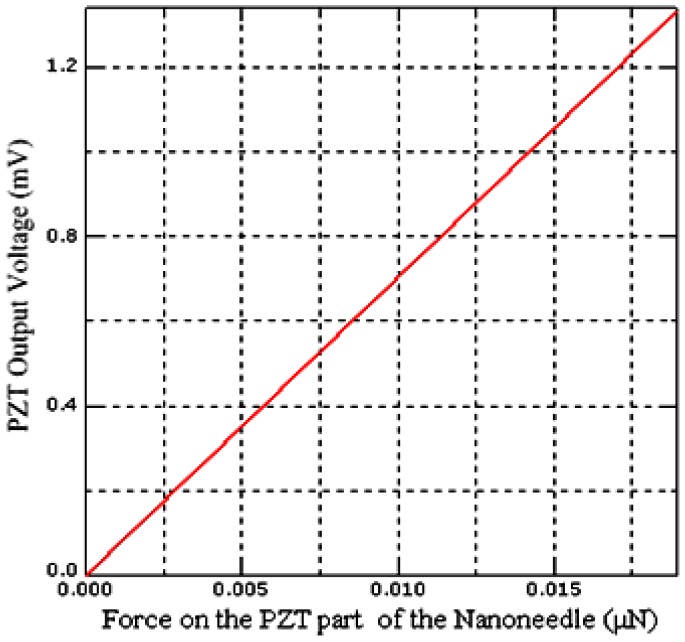
Voltage-force relationship of the PZT sensing part of the buckling nanoneedle.

When force is applied along the polarization axis of the PZT, Equation (2) is simplified to the axial PZT sensor equation as:

(3) $$Q=-d_{33}F$$

where, *Q* is the electrical charge generated, while *F* and *d_33_* are the force applied and piezoelectric charge coefficient, respectively. Figure 2 shows the relation between the applied force and the output voltage of the PZT. 

### 2.3. Finite Element Modelling of the Buckling Part of the Nanoneedle

The buckling part of the PZT-integrated nanoneedle serves as a suspension system to absorb any excessive force during indentation for single cell stiffness measurements. Cylindrical geometry with 95 nm radius and height of 9 µm was chosen for this part. The model has been built using 2D with desired dimensions and later transformed to 3D. Silicon was chosen as the material for the buckling part of the PZT-integrated buckling nanoneedle due to its flexibility and softness. The Young’s modulus (97.83 GPa) and Poisson’s ratio (0.22) of silicon were adopted from [4] and were used as the material properties of the buckling part of the PZT-integrated buckling nanoneedle. Silicon molecular density (2.39 g/cm^3^) adopted from [13] was used as the model density.

### 2.4. Integrating the PZT Sensor with the Buckling Part of the Nanoneedle

**Figure 3:**
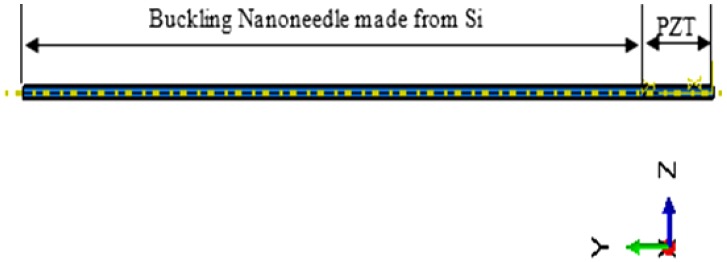
Finite element model of the composite buckling nanoneedle.

The sensor part of the nanoneedle and the buckling part are integrated together to form the complete PZT-integrated buckling nanoneedle. The sensor was integrated with the buckling part as shown in Figure 3. A tie constraint was applied for the two parts to behave as single composite material. As a result, the nanoneedle will have PZT properties as well as properties of silicon (Si). For this reason, it is expected that the stiffness of the composite buckling nanoneedle will be higher than that of the buckling nanoneedle part alone, due to the large value of the PZT material density.

Similarly, the Young’s modulus of the composite system is expected to be higher than that of a single material buckling needle, while the Poisson’s ratio may not be higher because of the uniform cross-sectional area of the two parts which will be investigated in the model calibration section.

### 2.5. Buckling Analysis of the PZT-Integrated Buckling Nanoneedle

The buckling phenomenon of the PZT-integrated buckling nanoneedle was analyzed numerically using the closed-form solutions proposed by Cheng et al. [15]. The solution of the applied force, during indentation process for an elastic solid using any desired shape *f*(*x*) stiff axisymmetric indenter, is given by Equation (4):

(4) $$F=\frac{4Ga}{1-\nu }\int_{0}^{1}\frac{x^{2}f^{'}(x)}{\sqrt{1-x^{2}}}dx$$

where *a* is the radius of the tip of the buckling nanoneedle that makes contact with a sample and *f’*(*x*) *= dF(x)*/*dx*. Assuming that, Passion’s ratio is independent of time, we can apply Lee and Radok theory to Equation (4). Hence, Equation (4) becomes:

(5) $$F(t)=\frac{4}{1-\nu }\int_{0}^{t}G(t-\tau )\frac{d}{d\tau }\left[a(\tau )\int_{0}^{1}\frac{x^{2}f^{'}(x)}{\sqrt{1-x^{2}}}dx\right]d\tau$$

Differentiating the equation using basic rules of differential calculus with respect to the time as reported by [15] with initial condition of constant unloading rate *ν_h_* after *t = t_m_* yields:

(6) dF(n)dh=dFdtdhdt=41−ν[G(0)a(tm)−1νh∫0tmdGdη|η=tm−ta(τ)dh(τ)dτdτ]

For fast unloading rate *ν_h_*, the second term of Equation (6) does not depend on the loading history. Hence, it can be approximated to zero. Therefore, Equation (6) becomes:

(7) $$\frac{dF}{dh}=\frac{4}{1-\nu }G(0)a(t_{m})=\frac{2E(0)}{1-\nu^{2}}{\left(Rh\right)}^{\frac{1}{2}}$$

where *E*(0) is the time dependent Young’s modulus of relaxation of the material.

Equation (7) was reported by [4]. The equation has not been validated experimentally due to some difficulties in using a standard AFM cantilever tip. For this reason, the buckling nanoneedle was used to validate the equation experimentally. The buckling nanoneedle spring behavior has the same directional axis as the applied force, however, the buckling behavior was assumed to be unidirectional, although the sample is not indented with negative force at the unloading stage during analysis as in the case of standard AFM. According to [4] experimental data from the buckling behavior of the nanoneedle tip has been used to determine the instantaneous relaxation elastic modulus. 

Ultimately, the force, *F*, in Equation (7) can be obtained using Euler’s equation for buckling as:

(8) $$F=\frac{E\pi^{2}I}{{\left(KL\right)}^{2}}$$

where, *F*, *E*, *I*, *K* and *L* are the force applied, Young’s modulus, second moment of inertia, effective length factor and effective length of the nanoneedle, respectively.

## 3. Calibration of PZT-Integrated Buckling Nanoneedle

The model was calibrated using the FE method and compared with the experimental method using nanomanipulation as proposed by [4] for validation purpose.

### 3.1. Experimental Method Using Nano Manipulation

In [4], the fabricated buckling nanoneedle was calibrated using a cantilever with a known spring constant (0.15 N·m^−1^), which has been determined experimentally. With the aid of nanomanipulator the fabricated buckling nanoneedle was used to indent the cantilever up to the time the buckling behavior begins to occur. The applied force on the nanoneedle was then removed until the needle returned to its original shape. The spring constant of the buckling nanoneedle was obtained as 0.45 N·m^−1^ by experiment from the linear region of the second slope of indentation force-buckle length of the nanoneedle [4]. The buckling force, *F_n_*, has been computed using Hooke’s law and is given by:

(9) $$F_{n}=k_{cantilever}\delta_{cantilever}$$

where, cantilever and *δ_cantilever_* are the spring constant and deflection of the cantilever from the point of reference, respectively. The Young’s modulus was calculated using Equation (8). Furthermore, the second moment of inertia, *I*, is given by Equation (10):

(10) $$I=\frac{\left(wb^{3}\right)}{12}$$

where, *w* and *b* are the width and length of the rectangular cross section of the fabricated nanoneedle, respectively. Using *K* as 0.8, because the nanoneedle was rigid at the interface between the base of the buckling nanoneedle and the nanomanipulator, while the other end was pinned [4] Equation (8) becomes:

(11) $$E_{nanoneedle}=\frac{7.68F_{n}l_{buckled}^{2}}{\pi^{2}wb^{3}}$$

Using as the dimensions of the fabricated buckling nanoneedle 190 nm × 210 nm for the cross sectional area and 10 μm as height, by applying Equation (11) the Young’s modulus was computed as 97.8 ± 2.1 GPa. This is in agreement with the value in the standard silicon parameters [4].

### 3.2. Calibration by the Finite Element Method

In this method, a 3D FE model of a rectangular cross section buckling nanoneedle was built using ABAQUS. The dimensions used are 190 nm × 210 nm for the cross sectional area and 10 μm as height [4]. Silicon material properties obtained by [4] using the nanomanipulation method have been used as the material properties of the model. These include a density of 2.32 × 10^−15^ kg·μm^−3^, Young’s modulus and Poisson’s ratio of 97.8 GPa and 0.22, respectively. The model was discretized by using good mesh to ensure accurate results during analysis. Appropriate constraints have been applied to the upper and lower nodes of the model. The upper nodes were unbound while the lower nodes has been allowed to translate along the y-axis only. To obtain the spring constant and Young’s modulus of the model using FE method two analysis are required: pre-buckling and post-buckling analysis. Pre-buckling uses a linear perturbation algorithm. During the analysis, a load of 0.2 μN was applied to the model along the y-axis. The solver estimated the eigenvalue (buckling load) based on the number of iteration and buckling mode for the result to converge. The eigenvalue extraction in the pre-buckling analysis was used to determine the stability of the structure by introducing some imperfections for post-buckling analysis. For the result to be available for the next analysis, the result has been saved using the keyword.

*NODE FILE
U

Post-buckling analysis uses a risk algorithm. In this analysis the indentation force and buckle length data were obtained based on the previous analysis with the same mesh using the keyword.

*IMPERFECTION, FILE = Pre-buckling_analysis, STEP = 1
1, 0.01

**Figure 4:**
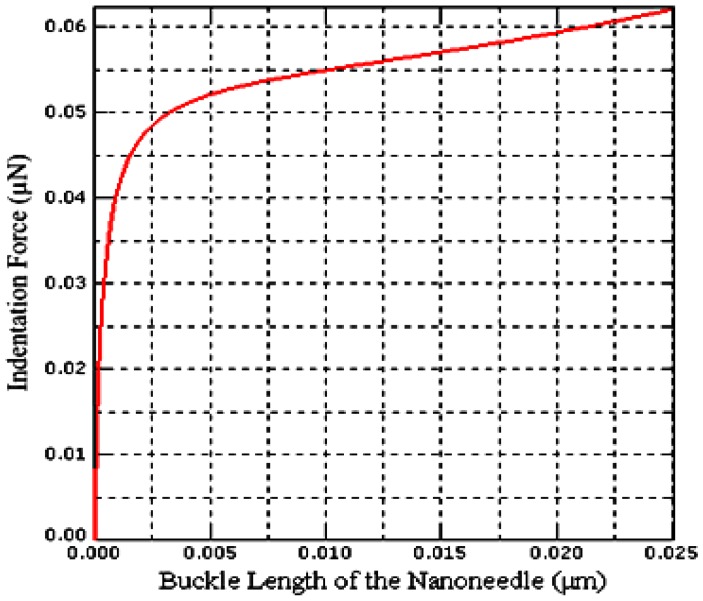
Indentation force-buckle length relationship of the rectangular buckling nanoneedle.

The spring constant of the model is 0.50 N·m^−1^ computed from the linear part of the second slope of indentation force- buckle length of the nanoneedle curve shown in Figure 4.

Additionally, using Equation (11), the Young’s modulus has been computed as 104 GPa. These results are in good agreement with the results obtained by the experimental method using nanomanipulation [4]. However, our interest is to calibrate the stiffness and Young’s modulus of cylindrical PZT-integrated buckling nanoneedle so that Hertz-Sneddon mechanics model can be used to measure single cell stiffness. The geometry of the previously built FE model has been changed to cylindrical with a radius of 95 nm and height of 10 µm. Despite the fact that the I for the rectangular nanoneedle in Equation (11) has been replaced with *I* for the cylindrical nanoneedle, the results are still in good agreement as shown in Table 2. 

For a cylindrical nanoneedle *I* is given by Equation (12):

(12) $$I=\frac{\pi r^{4}}{4}$$

where, *r* is the radius of the cross sectional area of the cylindrical nanoneedle.

Finally, the height of the FE model has been changed to 9 µm and integrated with PZT of the same radius and height of 1 µm. The result is still in good agreement with the experimental method result, as shown in Table 2.

**Figure 5:**
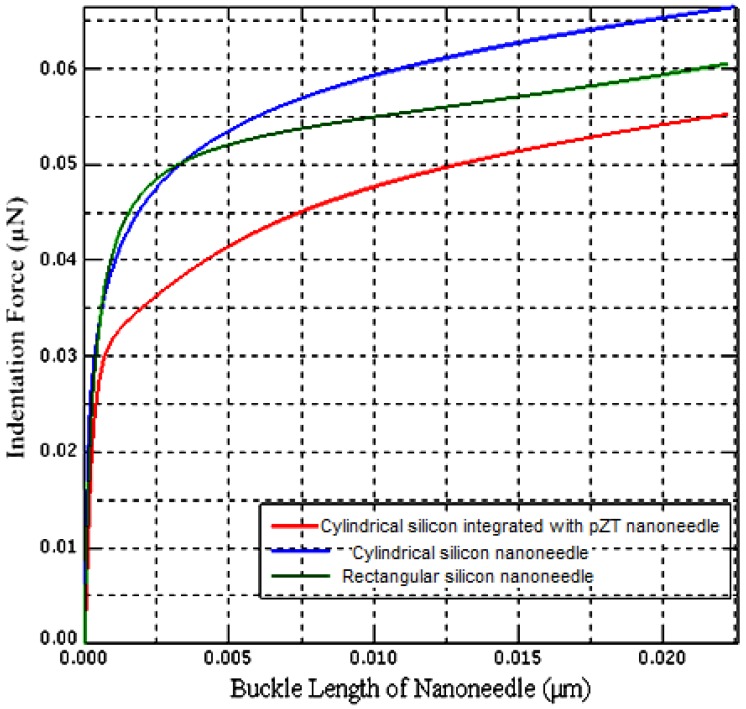
Comparison of the indentation force-buckle length relationship of the three different types of the buckling nanoneedle.

However, the slight variation of stiffness and Young’s modulus when the responses of the three buckling nanoneedles have been compared as shown in Figure 5 were because of the differences in geometry and material composition.

Poisson’s ratio of the integrated PZT with buckling nanoneedle was computed using a vcomposite material equation [26] given by Equation (13):

(13) $$\nu_{p-b}=\nu_{p}V_{p}+\nu_{s}(1-V_{p})$$

where *ν_p-b_*, *ν_p_*, *ν_s_* and *V_P_* are the Poisson’s ratio of the PZT-integrated buckling nanoneedle, Poisson’s ratio of the PZT, Poisson’s ratio of the buckling part, and ratio of the cross sectional area of the PZT part and the buckling part of the nanoneedle, respectively. 

Since both parts of the buckling nanoneedle have the same cross sectional area Equation (13) becomes:

(14) $$\nu_{p-b}=\nu_{p}V_{p}$$

From Equation (14), we can say that our PZT-integrated buckling nanoneedle Poisson’s ratio is approximately equal to the Poisson’s ratio of the PZT material as shown in Table 2.

We have investigated, during the loading and unloading phase PZT output voltage increases and decreases proportional to the loading and unloading force as shown in Figure 2. As a result, we obtained the sensitivity of the model by computing the slope of the voltage-force curve as 0.06933 V·m·N^−1^. The sensitivity value is in good agreement with the sensitivity value of PZT microfiber obtained by [27]. Indeed the result shows the effect of size on the sensitivity of the PZT material when comparing with the sensitivity of the bulk PZT material (0.025 V·m·N^−1^). However, at a micro level, the sensitivity of the PZT increased without change in output voltage, but fabrication is difficult [28,29,30]. From the model calibration parameters shown in Table 2, we could claim that our FE model is valid, because it is consistent with [27] and the buckling nanoneedle models proposed by [4].

## 4. Finite Element Modelling and Validation for *Saccharomyces cerevisiae* Yeast Cells

*Saccharomyces cerevisiae* material properties has been used for the FE sample cell model because of the similarities with higher animal (human) cells in terms of life cycle [11]. As a result, it is used for detection of regenerative diseases, for example cancer, fibroids and tumors in humans and studying numerous bioprocess fluids at the cell level [11,31].

However, *Saccharomyces cerevisiae* has a well-defined cell wall, which is contrary to human red blood cells (RBCs). Nevertheless, based on considerable assumptions, the *Saccharomyces cerevisiae* constitutive equation is applicable to RBCs because the problem of over parameterization is overcome and yields meaningful results [31,32]. In this paper, a *Saccharomyces cerevisiae* FE model has been built in ABAQUS with two different layers. The outer layer is the cell wall with a thickness of 90 nm adopted from [31] and the inner layer known as the cytoplasm. The diameter and height of the *Saccharomyces cerevisiae* FE model is 5 µm and 4.5 µm as reported by [4,11]. Young’s modulus, cytoplasm density, cell wall density and soft biological material Poisson’s ratio of 112 MPa, 1 g·mL^−1^, 1.084 × 10^−15^ kg·μm^−3^ and 0.5 respectively, has been used for the model as reported by [11,12,33]. To ensure solution convergence the Eulerian method has been used. The model was discretized by using a good mesh density to account for the non-linearity during analysis.

**Figure 6:**
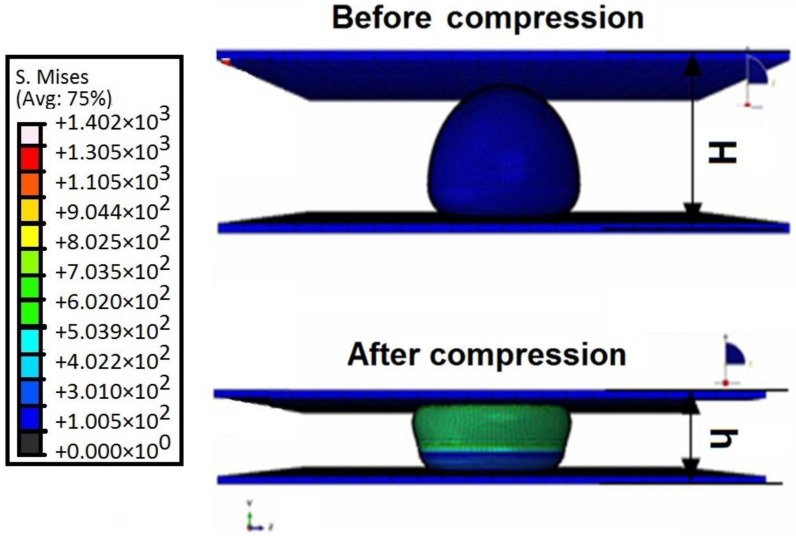
Cell validation using compression test.

Compression tests have been conducted to validate the model using experimental data obtained by [31,32,33,34,35,36,37]. In this test, the model is placed between two parallel rectangular plates as shows in Figure 6. A compression force has been applied to the model at a speed of 1.5 μm·s^−1^ which is in the range (1.03–7.68 μm·s^−1^) suggested by [32]. To extract the cell wall material properties we assumed that thickness of the cell wall before compression is uniform, incompressible and permeable. As a result, we have described the cell wall material properties of the model using constitutive linear isotropic elastic equation as [33]:

(15) $$\sigma =\left(1-\frac{E_{p}}{E}\right)\sigma_{y}+E_{p}\varepsilon$$

where *σ*, *σ_y_*, *E*, *E_p_* and *ε* are the uniaxial stress, elastic to plastic yield transition stress, Young’s, plastic modulus, and strain, respectively. 

Based on the assumption that, during the test Equation (15) is time independent. We can describe the fluid flow out of the cell using the Kedem-Katchalsky equation [32] given as:

(16) $$\frac{dV}{dt}=k_{t}A\left(\Delta P-\Delta p\right)$$

where, *V* defines instantaneous volume, *k_t_* is the permeability constant, *t* is time, Δ*P* is the change in hydrostatic, Δ*p* is the osmotic pressure and *A* is the available area for the flux.

**Figure 7:**
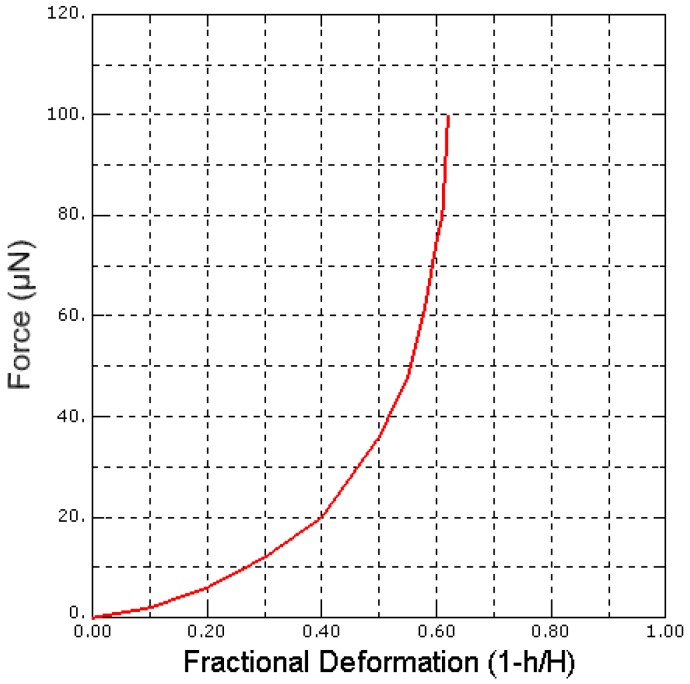
Force-fractional deformation curve of the cell.

ABAQUS software has been used to solve the formulated problem. Figure 7 shows the force-fractional deformation curve obtained from data generated by ABAQUS. From the curve we have computed the complete cell (global) stiffness of the *Saccharomyces cerevisiae* FE model to be 10.8867 ± 0.0094 N·m^−1^ and Young’s modulus of 110.7033 ± 0.0081 MPa under normal environmental conditions.

The result shows that our *Saccharomyces cerevisiae* FE model is valid, because the stiffness and the Young’s modulus obtained is in good agreement with the experimental result obtained by [31,32].

## 5. Finite Element Analysis of *Saccharomyces cerevisiae* Stiffness Measurement Using the PZT-Integrated Buckling Nanoneedle

**Figure 8:**
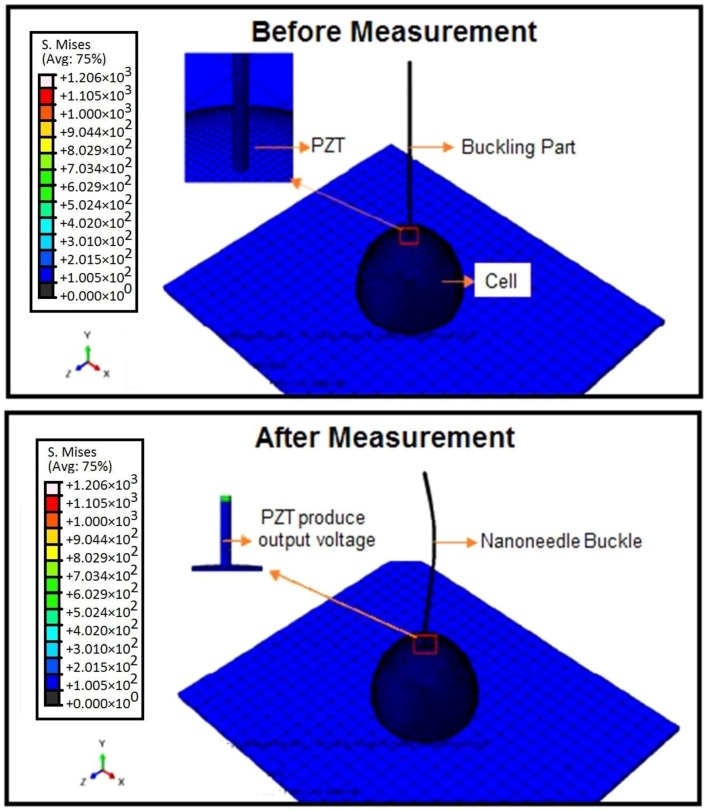
Cell stiffness measurement assembly in ABAQUS.

We have measured the global stiffness and Young’s modulus of a single *Saccharomyces cerevisiae* cell in the previous section. In this section local stiffness measurements of the FE cells models have been conducted. Figure 8. Shows the assembly of the PZT-integrated buckling nanoneedle and the *Saccharomyces cerevisiae* cell in ABAQUS for single cell local stiffness measurements. Different environmental conditions affect the stiffness of the cell [38]. For this reason, if the conditions change the stiffness of the cell to a very low value, virus and bacteria can penetrate the cell wall/membrane easily and attack the cell. Hence, the local stiffness have been investigated at different environmental conditions in the following subsections. 

### 5.1. Effect of Pressure on Local Stiffness Measurements of a Single Saccharomyces cerevisiae Cell

In this part of Section 5, the effect of environmental conditions such as pressure on the local stiffness of our FE *Saccharomyces cerevisiae* cell model has been investigated because variations in environmental conditions affect the physiological properties of living cell, for example, cell wall/membrane mechanics, for the cell to survive by adapting to the changes in the environmental conditions. The measurements have been done under two different conditions: 600 Pa and 0 °C (native conditions) and at 1.04 × 10^−3^ Pa (high vacuum conditions).

Under native conditions, the cell wall of *Saccharomyces cerevisiae* cells is still soft, because during measurements we observed that both the cell and the PZT-integrated buckling nanoneedle deform as shown in Figure 8. 

Using ABAQUS, the deformation of the buckling nanoneedle and the cell, PZT output voltage for three different size *Saccharomyces cerevisiae* cell models have been obtained. The parameters have been used to compute the local stiffness using equation [6] given as:

(17) $$k_{cell}=k_{needle}\left(\frac{\Delta_{total}-\Delta_{cell}}{\Delta_{cell}}\right)$$

where *k_cell_*, Δ*_cell_*, *k_needle_* and Δ*_total_* are the cell stiffness, deformation of the cell, needle stiffness and total deformation.

From Equation (17), the force on the surface of three different size cells models at the point of contact between the cell surface and the PZT-integrated buckling nanoneedle can be obtain using Hooke’s law, given as:

(18) $$F=k_{cell}\Delta_{cell}$$

where *F* defines the force on the cell surface, *k_cell_* defines the cell stiffness, and *Δ_cell_* is the deformation, of the cell. 

The Young’s modulus of the cells have been computed using the Hertz-Sneddon mechanics model [6] given as:

(19) $$F=\frac{2E_{cell}a\delta }{1-\upsilon^{2}}$$

where *F*, *E_cell_*, *ν*, *δ* and *a* are the force on the cell surface, Young’s modulus, Poisson’s ratio, indentation depth and tip radius of the nanoneedle, respectively. Table 3 shows that the results obtained under native conditions are in good agreement with the results reported by [4].

At 1.04 × 10^−3^ Pa (high vacuum conditions) most of the buckling nanoneedles fail to measure the stiffness of the cell because the cell is very hard and its deformation approaches zero. As a result, it is difficult or not possible to determine cell deformation. For this reason, Equation (17) fails. However, using the PZT-integrated buckling nanoneedle the stiffness can be obtained easily, which is one of the contributions of this paper, because we have shown previously in Section 2 of this paper that the PZT part of the buckling nanoneedle is linearly related to the force transmitted to the PZT. However, this force depends mainly on the stiffness of the cell under investigation. As a result, we compute the stiffness and Young’s modulus of the three different types of the cell model under high vacuum condition from the PZT output voltage as shown in Table 4. 

Under native conditions the average stiffness, Young’s modulus and PZT output voltage with their measure of dispersion from Table 3 are 0.4245 ± 0.0226 N/m, 1.6744 ± 0.0884 MPa, and 5.3502 ± 0.0349 nVolt, respectively. However, on the other hand, under high vacuum conditions, the PZT output voltage increases drastically. Similarly, the average stiffness, Young’s modulus and PZT output voltage with their measure of dispersion from Table 4 increase drastically to 4.2513 ± 0.0569 N/m, 16.7691 ± 0.2080 MPa, and 55.9221 ± 1.3787 nVolt, respectively. This is because the cell physiology changes for the cell to adapt to the changes in the environmental conditions.

### 5.2. Effect of Temperature on Local Stiffness Measurement of a Single Saccharomyces cerevisiae Cell

In this part, we have investigated the effect of temperature on the stiffness of our *Saccharomyces cerevisiae* cell model. The sample cell temperature has been increased from 0 °C to 3 °C then in steps of 2 °C up to 21 °C, while the pressure has been maintained at 600 Pa. The temperature has been increased from 0 °C to 3 °C so that the measurements can be validated with the experimental data obtained by [38] due to equipment limitations. 

**Figure 9:**
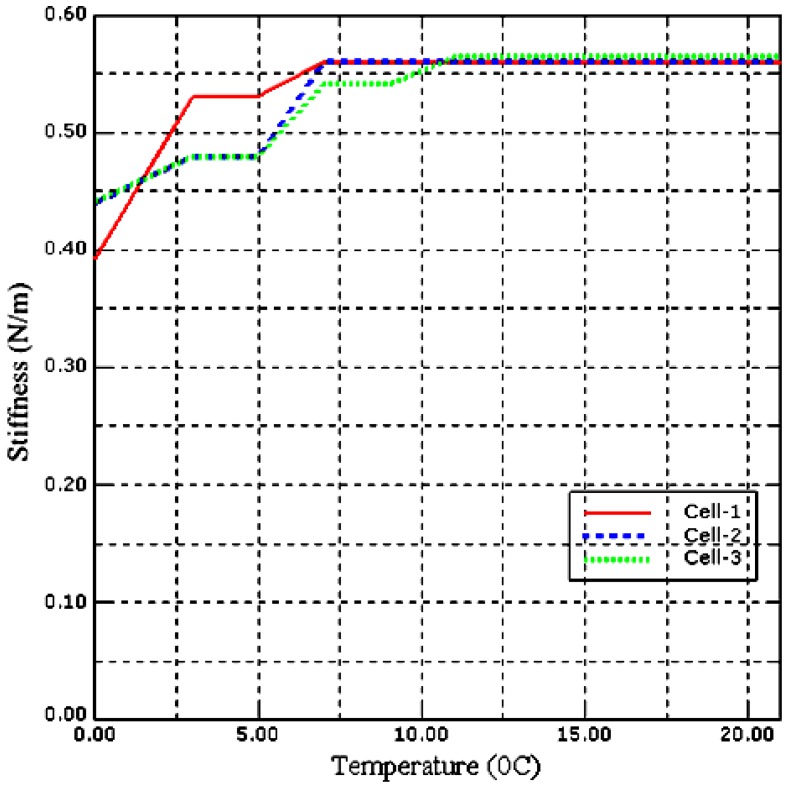
Stiffness of three different size yeast cells at different environmental temperatures.

**Figure 10:**
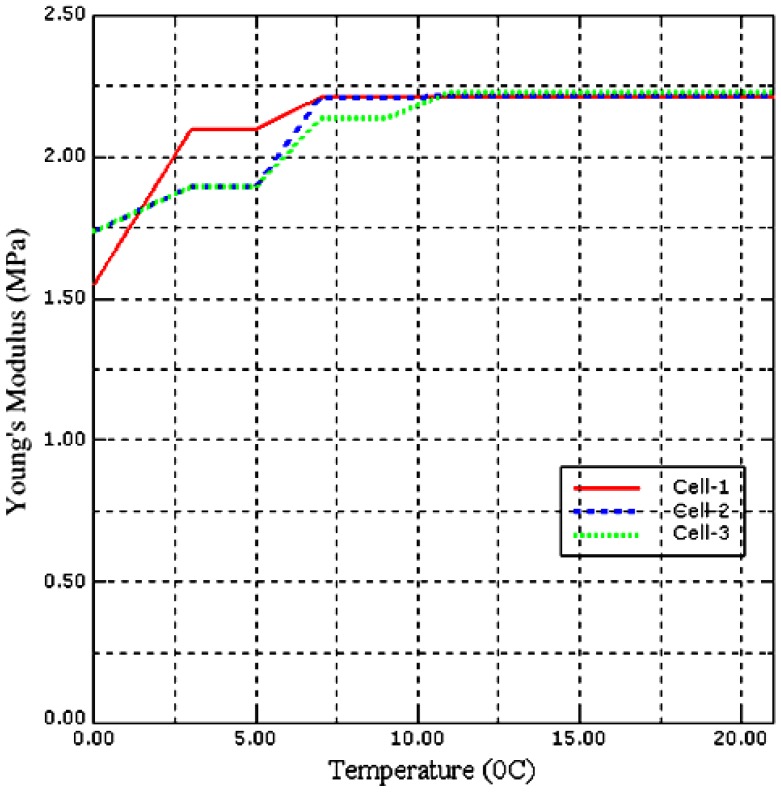
Young’s modulus of three different size yeast cells at different environmental temperatures.

At each temperature stiffness, the Young’s modulus and PZT output voltage have been obtained. Similar measurements have been conducted with two different size of the *Saccharomyces cerevisiae* cell models. Figure 9 and Figure 10 shows the stiffness-temperature and Young’s modulus-temperature curves obtained from data generated using ABAQUS.

From Figure 9 and Figure 10 the result is non-linear and shows how the stiffness and Young’s modulus of *Saccharomyces cerevisiae* varies with temperature and is in good agreement with the experimental results obtained by [38].

## 6. Conclusions

In this paper, we have demonstrated for the first time how to integrate PZT with a buckling nanoneedle for single cell stiffness measurements. Both the PZT and the buckling part of the nanoneedle were modelled separately and integrated together. The PZT-integrated buckling nanoneedle was calibrated, where the stiffness, Young’s modulus, Poisson’s ratio and sensitivity was obtained. *Saccharomyces cerevisiae* cells were successfully modelled and validated based on compression tests. We successfully assembled the PZT-integrated buckling nanoneedle and *Saccharomyces cerevisiae* cell for stiffness measurements. We investigated the stiffness of the cells under different environmental conditions. We successfully obtained the stiffness of the cell under high vacuum conditions. Under this condition, the existing buckling nanoneedles fail to measure the stiffness of the cell. Furthermore, we have shown that low temperature conditions are more dangerous to the cell because the stiffness becomes low where most of the viruses, bacteria and other various parasites can penetrate the cell wall/membrane and attack the cell easily. The advantage of this new technique is that it provides real time data measurement under different environmental conditions of the cell. Consequently, the technique will serve as a quick and accurate process to diagnosis diseases at early stages in a cell for effective treatment. These simulation results also motivate future work which will focus on experimental validation of the proposed system. Future work might include a laboratory experiment with the same proposed structure which is a PZT-integrated nanoneedle and a sensitivity analysis of the system.

## Figures and Tables

**Table 1:** PZT Parameters.

Category	Matrix of Parameters for Polarization Along y-Axis	Units
Compliance	$[\begin{array}{cccccc} 12.6 & 8.41 & 7.95 & 0 & 0 & 0\\ 8.41 & 11.7 & 8.41 & 0 & 0 & 0\\ 7.95 & 8.41 & 12.6 & 0 & 0 & 0\\ 0 & 0 & 0 & 2.3 & 0 & 0\\ 0 & 0 & 0 & 0 & 2.325 & 0\\ 0 & 0 & 0 & 0 & 0 & 2.3 \end{array}]\times 10^{-4}$	µN/µm^2^
Stress coefficient	$[\begin{array}{cccccc} 0 & 0 & 0 & 0 & 0 & 17\\ -6.5 & 23.3 & -6.5 & 0 & 0 & 0\\ 0 & 0 & 0 & 17 & 0 & 0 \end{array}]\times 10^{-12}$	C/µm^2^
Dielectric constants	$[\begin{array}{ccc} 1.505 & 0 & 0\\ 0 & 1.302 & 0\\ 0 & 0 & 1.505 \end{array}]\times 10^{-14}$	

**Table 2:** Calibration parameters.

Nanoneedle Geometry	Stiffness (N/m)	Young’s Modulus (GPa)	Poisson’s Ratio	PZT Sensitivity (V·m·N^−1^)
Rectangular	0.5000	104.0000	0.2200	No PZT
Cylindrical	0.4300	106.6900	0.2200	No PZT
Cylindrical integrated with PZT	0. 7100	123.4700	0.3000	0.0693

**Table 3:** Cell parameters measured under native conditions (0 °C and 600 Pa).

Sample	Stiffness (N/m)	Young’s Modulus (MPa)	Fcell (nN)	Voltage (nVolt)
Cell-1	0.3925	1.5493	77.7890	5.3931
Cell-2	0.4400	1.7370	80.1190	5.5546
Cell-3	0.4410	1.7369	73.6050	5.1030

**Table 4:** Cell parameters under high vacuum conditions (1.04 × 10^−3^ Pa).

Sample	Stiffness (N/m)	Young’s Modulus (MPa)	Fcell (µN)	Voltage (nVolt)
Cell-1	4.1877	16.5301	0.8103	57.5406
Cell-2	4.2404	16.7402	0.7851	54.1712
Cell-3	4.3257	17.0371	0.9034	56.0545
